# Modelling the Impact of Temperature-Induced Life History Plasticity and Mate Limitation on the Epidemic Potential of a Marine Ectoparasite

**DOI:** 10.1371/journal.pone.0088465

**Published:** 2014-02-05

**Authors:** Maya L. Groner, George Gettinby, Marit Stormoen, Crawford W. Revie, Ruth Cox

**Affiliations:** 1 Centre for Veterinary Epidemiological Research, Department of Health Management, Atlantic Veterinary College, University of Prince Edward Island, Charlottetown, Prince Edward Island, Canada; 2 Department of Mathematics and Statistics, University of Strathclyde, Glasgow, Scotland, United Kingdom; 3 Centre of Epidemiology and Biostatistics, Norwegian School of Veterinary Science, Oslo, Norway; College of Charleston, United States of America

## Abstract

Temperature is hypothesized to contribute to increased pathogenicity and virulence of many marine diseases. The sea louse (*Lepeophtheirus salmonis*) is an ectoparasite of salmonids that exhibits strong life-history plasticity in response to temperature; however, the effect of temperature on the epidemiology of this parasite has not been rigorously examined. We used matrix population modelling to examine the influence of temperature on demographic parameters of sea lice parasitizing farmed salmon. Demographically-stochastic population projection matrices were created using parameters from the existing literature on vital rates of sea lice at different fixed temperatures and yearly temperature profiles. In addition, we quantified the effectiveness of a single stage-specific control applied at different times during a year with seasonal temperature changes. We found that the epidemic potential of sea lice increased with temperature due to a decrease in generation time and an increase in the net reproductive rate. In addition, mate limitation constrained population growth more at low temperatures than at high temperatures. Our model predicts that control measures targeting preadults and chalimus are most effective regardless of the temperature. The predictions from this model suggest that temperature can dramatically change vital rates of sea lice and can increase population growth. The results of this study suggest that sea surface temperatures should be considered when choosing salmon farm sites and designing management plans to control sea louse infestations. More broadly, this study demonstrates the utility of matrix population modelling for epidemiological studies.

## Introduction

Many marine pathogens are capable of causing dramatic population-, community- and ecosystem-level shifts and the patterns of infection are frequently associated with temperature [1], [2], [3], [4], [5]. In particular, high temperatures are often associated with increased frequency or severity of infection, as a result of altered development and survival of the pathogen, physiological changes in the host and range expansions [1], [2], [6], [7], [8]. Understanding the role that temperature plays in the epidemiology of marine diseases is important for predicting and potentially mitigating infestations and may be important for forecasting disease risk in a climate change context.

Quantifying the influence of temperature on infections in marine environments is challenging. For many marine pathosystems, there is a lack of baseline data on how temperature influences epidemiological patterns and those that exist are often confounded with other influential water quality information (e.g., salinity, circulation) [1], [4]. In addition, temperature can influence the host and the pathogen separately, and these effects may differ among life history stages. In many cases only some of these interactions are understood or the etiologic agent of disease is unknown [1], [2], [6], [9]. Despite these challenges, water temperature often follows well-defined seasonal patterns and its effects should be predictable.

Open-pen aquaculture may offer a unique opportunity to understand the role of temperature on marine diseases. In particular, because these systems often control spatial and temporal variation in host densities, they can be used to examine the role of temperature in influencing pathogen life history and virulence. One case where temperature may be especially influential is that of sea louse (*Lepeophtheirus salmonis*) infestations on salmonids. Sea lice are an ectoparasite of farmed and wild salmonids (Atlantic salmon (*Salmo salar*), steelhead (*Oncorhynchus mykiss*), and Pacific salmon (*Oncorhynchus* spp.)) and infestations have been associated with declines in returns of adult wild salmonids [10], [11], [12]. Sea lice have very plastic life history responses to temperature. For example, the generation time of sea lice has been estimated to range between 50 days at 12°C and 114 days at 7°C [13], suggesting that infestations may increase in response to warmer temperatures. Nonetheless, the role that temperature plays in sea louse infestations is not clear. While controlled laboratory manipulations consistently find strong effects of temperature on sea louse development [14], effects of temperature on the population dynamics of sea lice are only detectable in some field data [15], [16], [17].

Many fish farms experience substantial economic losses due to morbidity of infested stock as well as the use of expensive chemotherapeutants to control sea lice [18]. A number of methods have been pursued within the salmon industry to control sea lice infestations on farms. These include adoption of integrated pest management approaches in which management areas, defined by hydrological boundaries, are fallowed periodically to break the sea lice reinfection cycle and all salmon in the management area are restricted to a single age cohort to avoid infection between age-classes. In addition to these practices, chemotherapeutant treatments are often necessary to control sea lice [19], [20]. While they have the potential to be very effective at reducing densities of attached sea lice (chalimus and mobiles) [21], [22], the success rates of chemical treatments often vary and in some cases numerous treatments are required to control sea louse populations [20]. Additional concerns with chemical treatments arise because they can be expensive [18], are stressful to salmon [23], have potentially detrimental environmental impacts [24], can be hazardous to the workers that dispense them and are proving to be less effective over time because sea lice have evolved resistance [20] [25]. Different treatments target different stages of sea lice and while both temperature and the stage targeted may influence the efficacy of a treatment, the role of these factors has not been throroughly investigated.

A range of modelling techniques have been used to evaluate sea louse population growth over time, including delay differential equation models [26], [27], individual-based models [28], advection-diffusion models [29], system dynamic models [30], and stochastic Monte Carlo simulation models [31]. While many of these models include temperature, we are only aware of one that has explicitly examined the effect of temperature variation on population demography and vital rates [14].

Matrix population models provide a useful tool for exploring the interactions between life history, temperature and population demography. Matrix population models can be manipulated to incorporate life history variation, stochasticity, environmental-dependencies and population feedbacks (e.g. density dependence) [32]. Moreover, analytical tools are well-developed for understanding the contribution of all of these factors to population demographics [32]. For example, elasticity analysis can be used to examine the effect of proportional changes to contributions of life stages (defined as matrix elements) on population growth, while sensitivity analysis can be used to examine the effect of absolute changes in life stage properties on population growth. The elasticities of population growth to changes in matrix elements can be used to predict the effectiveness of stage-targeted control methods, while the sensitivities of population growth to changes in matrix elements can provide insight in predicting how a population will evolve in response to selection at a specific life stage [32]. Comparison of elasticities and sensitivities of matrices constructed for the same organism at different temperatures can be used to understand how temperature-induced life history plasticity may alter population demographics. While population matrix models have a long history of use in conservation biology and pest management [33], [34], [35], they have rarely been used to understand the epidemiology of marine pathogens or parasites [36].

In this study we use stochastic matrix population models to understand the influence of temperature on the population growth, reproduction and demography of sea lice (*L. salmonis*) on farmed Atlantic salmon. We use sensitivity and elasticity analyses to understand the contribution of each life stage to population growth. We also examine how density-dependent mating and the rate that larval sea louse attach to hosts influence these patterns. Finally we evaluate these results in terms of the effect of temperature on population growth and effective control of sea lice.

## Materials and Methods

### Matrix Construction

To evaluate the effects of temperature, seasonality and the host attachment rate on sea louse demography, we created stage-structured population projection matrices (PPM) for female sea lice based on parameters from the literature. The model does not explicitly include Atlantic salmon hosts because they are not expected to influence the epidemiology of sea lice. This is because they exhibit little immune response to sea lice [37] and are maintained at constant densities throughout the salt water production phase.

**Figure 1:**
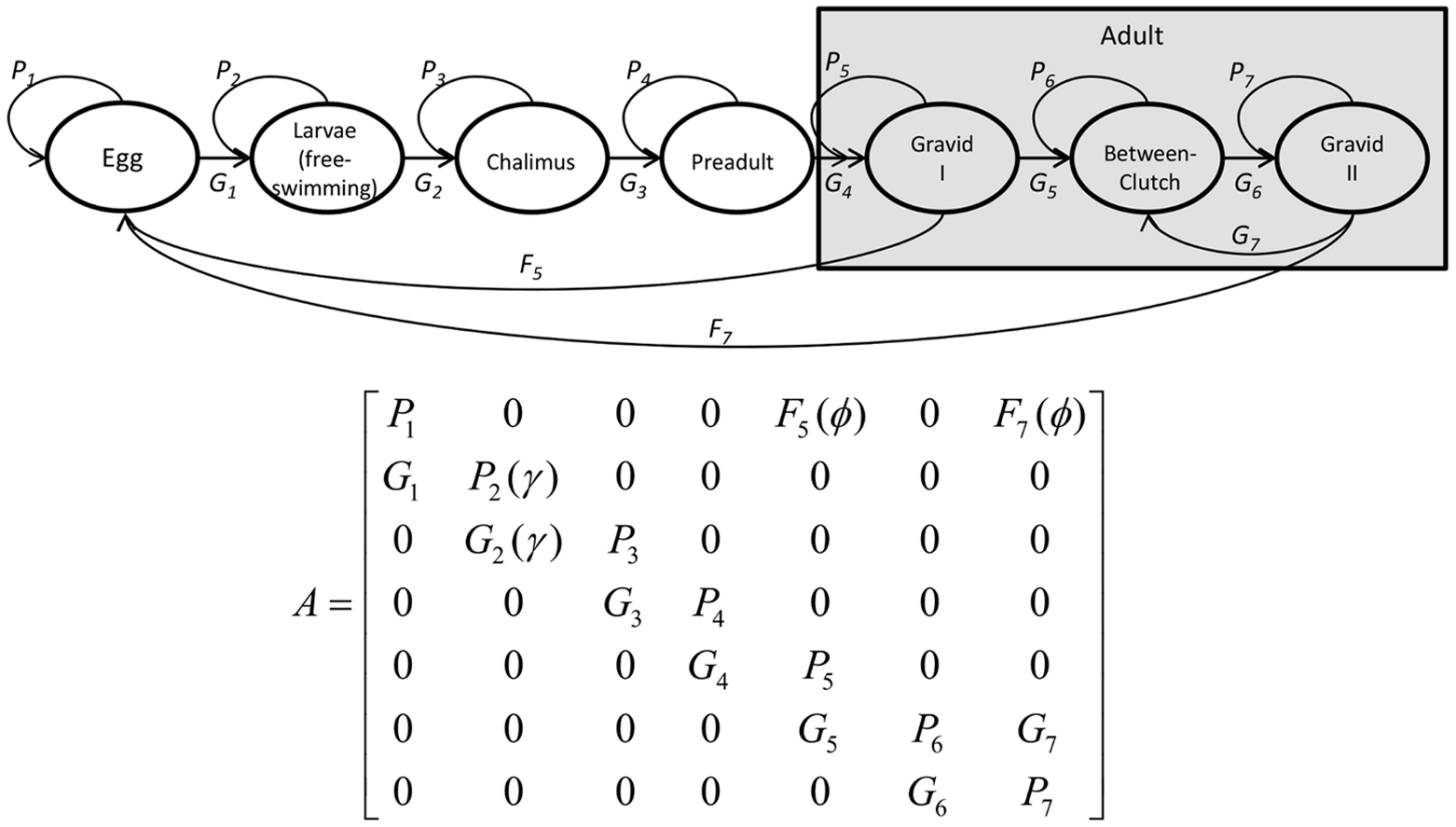
Diagram of stage-structured population projection matrix that is used in simulations. *P_i_* indicates the probability of staying in a stage, *G_i_* indicates the probability of transitioning to another stage and *F_i_* indicates fecundity. Survival and attachment of free-swimming larvae are a function of the rate that they attach to hosts (*γ*) and fecundity is a function of the probability of mating (*φ*).

*L. salmonis* transition through nine recognised life stages [38]. After hatching from the egg, the sea louse goes through three unattached stages during which it does not feed: nauplii (2 stages) and copepodid. Once the copepodid finds a host, it develops through two chalimus stages, two preadult and one adult stage. In our model, we reduced the life cycle to seven stages that reflect biologically important transitions: egg, larvae (consisting of nauplii I and II and copepodid), chalimus (stages I and II), preadult (I and II) and three adult phases which will be referred to as gravid I, between-clutch and gravid II (Figure 1). The three adult phases are separated here because they differ in terms of fecundity. Transitions from stage to stage occur in one direction, with the exception that females can transition from gravid II to between-clutch, and then back to gravid II, reflecting observations that females can produce up to 11 successive pairs of egg strings [39]. The population projection matrix represents daily transitions and operates on the life stage state vector with elements [egg, larvae, chalimus, preadult, gravid I, between-clutch and gravid II] (Figure 1).

Entries on the diagonal (*P_i_*) indicate the proportion of individuals remaining in a stage, entries on the sub- and super-diagonal (*G_i_*) indicate the proportion of individuals developing into a new stage and *F_5_* and *F_7_* indicate the fecundity of gravid I and gravid II adult females, respectively. *P_2_* and *G_2_* are a function of the rate that sea lice attach to the host (γ, described below), and *G_5_* and *G_6_* and *G_7_* are a function of egg hatching and development (described below). The remaining *P_i_* and *G_i_* elements, together with the *F_i_* elements, are defined as shown:
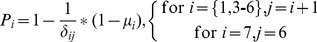
(1)

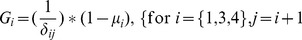
(2)

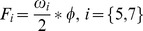
(3)where *δ_i j_ = *time to develop from stage *i* to stage *j, µ_i_ = *mortality rate at stage *i*, and *ω_i_* = number of viable eggs in the clutch produced at stage *i.* This number is multiplied by the probability of mating (*φ*) and divided by two because the matrix only considers females and assumes a 1∶1 sex ratio.

### Parameters

#### Developmental transitions

With the exception of copepodids, developmental transitions of sea lice were temperature-dependent. We parameterized developmental times in our model based on a review by Stien et al. 2005 [14] of existing data on temperature-dependent development in *L. salmonis* (Table 1). This review uses developmental rates across a temperature range to parameterize a modified Belahrádek equation [40]:

**Table 1:** Parameters used for matrix model calculations (equations 1–8).

Life Stage	*β_1_* (Standard Error)	*β_ 2_* (Standard Error)	ν
Eggs	41.98 (2.85)	0.338 (0.012)	2
Nauplii	24.79 (1.43)	0.525 (0.017)	0
Chalimus	74.7 (33.64)	0.236 (0.007)	0.85
Pre-adult Female	67.47 (20.36)	0.177 (0.006)	0.34

Parameters used in equations 4 and 5 to estimate developmental rates of each stage (from [14]).



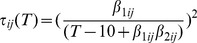
(4)where *τ_ij_* is the minimum required developmental time for individual *i* in stage *j* at temperature *T*. *β_1ij_* is a shape parameter and *β_2ij_^−^* ^2^ is the average for τ at 10°C. Variation in developmental rates was incorporated by randomly selecting values for *β_1ij_* and *β_2ij_* from a normal distribution with mean and standard deviation taken from [14]. A stage-specific constant, ν_j_, was added to this temperature-dependent estimate, to represent additional time beyond the minimum developmental time needed to make developmental transitions. Total developmental time (*δ_ij_*) of individual *i* at stage *j* is:



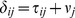
(5)Values of ν were from [14] and the developmental rates were the inverse of the developmental times. There is no evidence that copepodid developmental rates are temperature-dependent, so all copepodids in this model developed in 4.6 days, the mean value as estimated by [14]. The developmental rate of larvae was calculated as the inverse of the sum of nauplii and copepodid developmental times.

#### Survival

Survival estimates (1-*µ_i_*) for all stages except larvae were stochastically drawn from a triangular distribution defined by the minimum, maximum and most probable survival times based on data from [41] (Table 2). As with many invertebrates, there is little evidence that survival of sea lice is directly dependent upon temperature [14], [26].

**Table 2:** Survival rates for each life stage of the sea louse.

Parameters	Mean	Lower	Upper	Source
Egg Viability	0.90	0.75	0.96	[39]
Nauplii Survival (daily)	0.83			[14]
Chalimus Survival(daily)	0.992	0.98	0.997	[41]
Preadult Survival(daily)	0.965	0.953	0.98	[41]
Adult Survival (daily)	0.965	0.904	0.997	[41]

With the exception of nauplii, survival estimates for individuals were randomly drawn from a triangular distribution with lower and upper values shown.

Survival of larvae was the product of nauplii survival (calculated as above) and copepodid survival. Survival of copepodids depends upon the attachment of copepodids to a host, a process which is sensitive to local host densities and abiotic factors including currents and salinities [15], [17], [42]. Daily survival rates of copepodids (*S_c_*) depend on the attachment rate (*γ*) as well as the development time (*δ_c_*) such that:
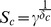
(6)


Since the attachment rate of copepodids varies considerably in nature, we simulated scenarios with several different values for *γ* (described in *Analyses*).

#### Fecundity

Sea lice reproduce sexually and females have two external egg strings in each clutch that are attached to them until hatching. Egg string production and hatching are synchronized on an individual louse. Estimates of egg viability, clutch size and time to hatching were based on data from [39]. Sea lice are estimated to have 152 ± 31 (mean ± SD) eggs per egg string in the first clutch and 296 ± 100 eggs per egg string in subsequent clutches [39]. Estimates of clutch size were chosen from these normal distributions and multiplied by two to account for both egg strings. The number of eggs produced was then multiplied by the estimated viability (Tables 1 and 2) and divided by two because the model only tracks female members of the population. There is little evidence for an effect of temperature or clutch order on egg viability [39].

Because the first clutch of eggs is substantially smaller than subsequent clutches we divided adult female stages into three parts, gravid I to represent the first extrusion of eggs (represented by *F_5_*), gravid II to represent subsequent extrusions of eggs (represented by *F_7_*) and between-clutch to represent the time between the extrusion of eggs. After completing the gravid I stage (*P_5_*), individuals will alternate between the between-clutch and gravid II stages represented by *P_6_*, *P_7_*, *G_6_* and *G_7_* in the PPM.

The time spent between clutches was the sum of the time needed for eggs to develop after egg string extrusion (*δ_egg_*-1) and the time between the hatching of one clutch and the release of the next egg string (*ζ*). Both of these are temperature-dependent. The latter parameter was estimated with the following relationship:
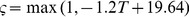
(7)


This equation is based on hatching times at 7.2°C and 12.2°C [39] and the observation that the shortest time between clutches, which is observed at temperature > 15°C, is ∼24 hours [43]. Egg extrusion 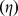
(e.g., the time in the gravid I or gravid II stages) was set at one day [43]. *G_5_*, *G_6_* and *G_7_* are therefore defined as:
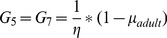
(8)

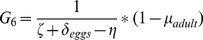
(9)


#### Density-dependence

Recent models of sea louse mating suggest that reproduction in sea lice is limited by mate availability when the abundance of sea lice is low [31], [44]. We incorporated this density-dependent effect, also called depensation or an Allee effect [32], in some iterations of the model by reducing the fecundity by the probability of mating (φ), which was calculated based on the ratio of adult sea lice to hosts.

To include density-dependent mating in our model, we used a variation of the model presented by [45]. The model assumes parasites are distributed on hosts according to the negative binomial distribution. This distribution is suited for dioecious parasites that aggregate together. It simulates the probability that a female will mate (φ) as a function of the mean number of adult lice on a host (*m,* calculated here as twice the number of adult females, therefore assuming and equal sex ratio) and a parameter describing overdispersion of sea lice among hosts (*k*) such that:
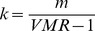
(10)where *VMR* is the variance to mean ratio of adult sea lice on hosts. Because sea lice are polygamous [46], we used a variation of this model that assumes that parasites coaggregate and that mating occurs for all females when there is at least one male on a host (i.e. complete promiscuity):
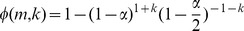
(11)where α = m/(m+k). The model assumes an equal sex ratio [47]. In the special case where 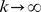, VMR = 1 and the lice assume a Poisson distribution among hosts with probability of mating simplifying to:



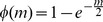
(12)For models where this density-dependent effect was included, fecundity estimates *F_5_* and *F_7_*, were multiplied by *φ* .

### Analyses

We calculated PPMs for a number of relevant scenarios, including a range of fixed temperatures and larval attachment rates, density-dependent mating, and yearly temperature profiles (described below). Depending upon the scenario, we examined some or all of the following demographic parameters: population growth (λ), reproductive rate (R_0_), generation time, and the sensitivity and elasticity of population growth to changes in matrix elements.

In our equations, λ was equal to the rate of population change over a day (i.e. the dominant eigenvalue of the matrix **A**). R_0_ was equal to the average number of offspring by which an egg will be replaced within its lifetime (i.e. the rate by which the population increases from one generation to the next) and generation time was equal to the time necessary to produce the number of offspring predicted by R_0_ [32]. Population growth is stable when λ = 1, decreases for λ < 1 and increases for λ > 1. Sensitivity was calculated as the effect of absolute changes to matrix elements on the population growth rate,
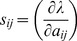
(13)


while elasticities were calculated as the effect of proportional changes in matrix elements on the population growth rate,
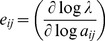
(14)where *a_ij_* indicates the matrix element [32].

In addition to the above analyses we performed a number of simulations to examine how various starting conditions affected population dynamics. All analyses were implemented in R (*v.* 2.15.0) using the ‘popbio’ package [48]. Details about how stochastic effects were included into each analysis are included below. Matrices showing means and standard deviations for all fixed temperature scenarios are in supplemental appendix S1. Annotated R-code is available in supplemental code S1, S2, S3, S4, S5, S6, S7 and S8.

#### Effects of fixed temperatures and larval attachment rates

In order to understand the effect of temperature on population growth rate, we calculated the matrix **A** for the following water temperatures, 4°C, 8°C, 12°C, 16°C and 20°C. These temperatures are within the range typically experienced by sea lice [49]. While temperatures colder than 4°C are also likely to occur in some locations (e.g. [17]), there are no data available to parameterize life history traits at these values.

The proportion of copepodids that attach to a host varies considerably in nature as a function of host behaviour and water salinity, hydrodynamics and light availability [50], [51], [52]. While there is some evidence that temperature may influence attachment rates of copepodids, it was not conclusive enough to include in the model [50], [51]. In order to measure how this variation influences sea lice populations, for each specific temperature matrix, we calculated the matrix **A** with different values for the attachment rate (*γ = *0.001, 0.01, 0.1, 0.5, 0.9), at each of the fixed temperature profiles.

Fixed temperature scenarios included demographic stochasticity where variation can be estimated from the literature. Individual louse developmental, survival and fecundity estimates in the PPM were calculated independently in each run (see descriptions above) and clutch sizes were drawn from normal distributions (described in *developmental transitions*). Survival estimates were drawn from triangular distributions (described in *survival*). For all fixed scenarios, we calculated the matrix **A** 1000 times to create a distribution of matrices which we used to calculate the mean and 95% confidence intervals for the intrinsic population growth rate, the net reproductive rate and the generation time.

We calculated the sensitivity and elasticity of λ to matrix elements. Graphical displays of these results show the sums of elasticities or sensitivities associated with a life stage. For example, the sensitivity of λ to larval sea lice is the sum of the sensitivity of *G_2_* and *P_2_* and the elasticity of λ to fecundity is the sum of the elasticities of λ to *F_5_* and *F_7_*. Matrices of means and standard deviations for elasticities and sensitivities in all fixed temperature scenarios and larval attachment rates are shown in supplemental appendix S2 and S3.

See supplemental appendix S4 for calculations of the proportion of individuals at each life stage at equilibrium and simulations to determine the time until equilibrium was reached (Figures S1 and S2 in supplemental appendix S4).

#### Effects of temperature on density-dependent mating

Density-dependent effects on population growth result in nonlinear models and the analyses described above are therefore not applicable. In order to understand how temperature and the larval attachment rate described above influence density-dependent mating, we ran simulations using density-dependent terms for fecundity elements *F_5_* and *F_7_* of the projection matrix. In each simulation we began with a population of 0.1 adult gravid I females per host. The model was simulated at the constant temperatures described above until the threshold of 3 adult female lice per host was crossed. This threshold was chosen because at this abundance mating success is nearly 95% for all models (4A) and mate limitation begins to have negligible effect on population growth. This was simulated by calculating matrix values associated with each life stage 100 times and using the means of these values for *VMR* = 1, 1.3 and 2 as well as in cases where density-dependent mating was not modelled.

#### Effects of seasonality

Understanding the demographic properties of sea louse populations at a single temperature is useful for developing a conceptual understanding of the effect of temperature; however, sea lice live in environments that typically experience substantial seasonal temperature variation. It is unclear how variation from ‘typical’ seasonal patterns might influence population growth rates. Therefore, we evaluated the intrinsic population growth rate across seasons for a variety of temperature scenarios. The baseline temperature profile was a sine curve fitted to a mean of temperature data collected at 33 fish farm sites in Scotland over a 5 year period [53]. We varied this temperature profile to create the following scenarios: cold and warm years (all temperatures 2°C below or above baseline), a year with cold winters (winter minimum is 2°C below baseline), a year with warm summer (summer maximum is 2°C above baseline), a year of ‘more seasonal’ temperatures (same mean temperature, but minimum and maximum temperatures are 2 degrees more extreme than in the baseline scenario) and a year of ‘most seasonal’ temperatures (same mean temperature, but the minimum winter and maximum summer temperatures are 4.5°C more extreme than in the baseline scenario). In all seasonal scenarios, we started the simulation in spring (i.e. day 1 began 120 days into the calendar year).

We calculated yearly PPMs for each temperature profile and each of five larval attachment rates (*γ = *0.001, 0.01, 0.1, 0.5, 0.9). To do this we constructed a PPM for each day (***B*** *_day_*) based on *γ* and the predicted temperature for that day. An overall PPM for the year was calculated by multiplying PPMs such that:
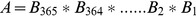
(15)


This process was repeated 1000 times and the intrinsic population growth (λ_daily_ ± 95% confidence intervals) was calculated from the resulting **A** matrices.

#### Effects of treating different life stages across seasons

Chemical and biological treatments to control sea lice target different life stages are used in response to elevated sea louse abundances. Instead of simulating specific types of treatments as has been attempted in previous models (e.g. [27], [28]) we model a generic treatment that will provide insight as to how temperature and the stage being targeted influence the effect of a treatment on λ_daily_. To do this, we create a treatment matrix, **H**, which is the identity matrix, with the exception that targeted stages are 1-*e*, where *e* is the efficacy of the treatment. For example, this matrix targets all adults:
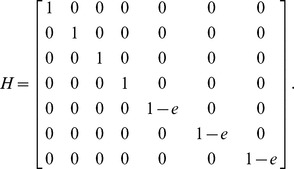



This matrix is then incorporated into the most extreme seasonal temperature matrices (described above) such that:
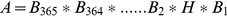
(16)where **H** is inserted when the treatment should occur. Scenarios were run in which a single treatment targeting one or combinations of several life-stages was delivered between day 1 and day 365.

The efficacy of treatments varies considerably from farm to farm. For example, the field efficacy of emamectin benzoate has been estimated to be anywhere between 60 and 99% in naive populations [54], but may be considerably less in resistant populations [55]. For the purposes of this study we set *e = *0.95. Because we expect that analyses performed with lower efficacy will have qualitatively similar results, we only explore one value for *e*.

For these analyses, we quantified mean effects on population growth. To construct matrices, we quantified 1000 development times, survival estimates and (where relevant) fecundity and viability estimates and used the mean values in these distributions to calculate **B**_day_ matrices.

## Results

### Effects of Fixed Temperatures and Larval Attachment Rates

**Figure 2:**
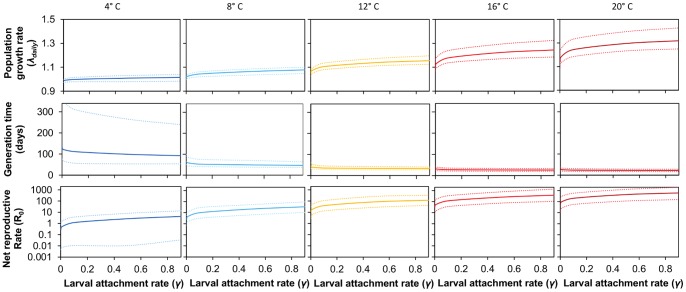
Effects of temperature and larval attachment rate on λ, R_0_ and generation time. Dotted lines indicate 95% confidence intervals. Population growth is positive when λ is greater than 1.

Increasing temperature caused λ and R_0_ to increase and generation time of sea lice to decrease (Figure 2), though the extent of these changes depended upon the larval attachment rate. Both temperature and the larval attachment rate caused λ to increase. When *γ* = 0.001, λ_daily_ was 0.99 at 4°C and 1.14 at 20°C. When *γ* = 0.5, λ_daily_ was 1.013 at 4°C and 1.28 at 20°C. The generation time of sea lice was between three and four times longer at 4°C compared to 20°C and increased with lower values of *γ*. For example, when *γ* = 0.001, the generation time was 107 days at 4°C and 28 days at 20°C. When *γ* = 0.5, the generation time was 73 days at 4°C and 23 days at 20°C. The generation time was longer with lower values of *γ*. R_0_ was greatest when the larval attachment rate and temperature were high. For example, when *γ* = 0.001, R_0_ increased from 0.25 at 4°C to 38 at 20°C. When *γ* = 0.5, R_0_ increased from 2.6 at 4°C to 321 at 20°C.

**Figure 3:**
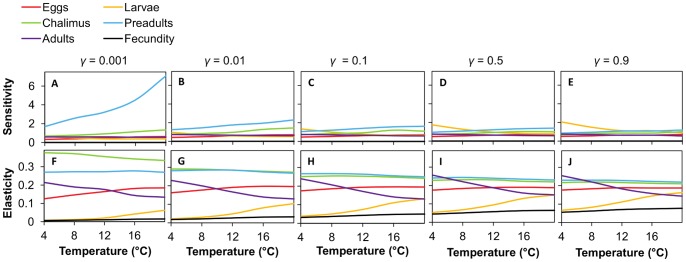
Sensitivities and elasticities of population growth rate (λ) to matrix elements. The sum of the elasticities of matrix values for surviving in (*P_i_*) and transitioning out of (*G_i_*) the same stages are presented.

Sensitivity analysis showed that, in general, λ is most sensitive to the survival and development of preadults (Figure 3).Sensitivity to this life stage is greatest when the attachment rate is low and the temperature is high. At high attachment rates and low temperatures, λ is most sensitive to the survival and development of larval sea lice.

Elasticity analysis shows that λ is most sensitive to proportional changes in matrix elements associated preadults and chalimus (Figure 3). The elasticity values associated with these terms decrease slightly with an increasing larval attachment rate and are relatively insensitive to temperature.

### Density-dependent Results

**Figure 4:**
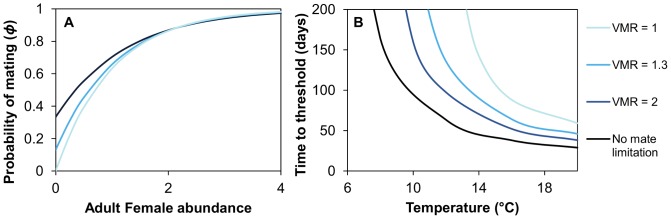
Effect of temperature on population growth when mate limitation is included. Figure A shows the probability of mating as a function of the abundance of adult females per host. Figure B shows the time to reach the threshold abundance of three female lice per fish (at which point mate-limitation is negligible) when sea lice abundances on hosts have a variance to mean ratio (*VMR*) of 1, 1.3 and 2. For all scenarios, the initial fish lice density was 0.1 fish per host and the attachment rate of larvae to the host was 0.1.

The probability of mating increases with the abundance of adult female lice (Figure 4A). The rate of this change is much faster when parasites are aggregated. When adult female lice are at low abundances the probability of mating increases when females are aggregated; however, above an abundance of one, aggregation has little effect on the probability of mating. Above abundances of three adult female lice, the probability of mating approaches 1.

The time necessary to reach the depensation threshold of three adult female lice per fish is shortest when lice are aggregated and the temperature is high (Figure 4B). At 20°C, a population of lice with an initial abundance of 0.1 adult females per louse will reach the threshold in 30 days for *VMR = *2 and 44 days for *VMR = *1. At colder temperatures the population growth is so slow that sea lice will take over a year to reach the depensation threshold. This occurs at 5°C when *VMR* = 2 and at 10°C when *VMR = *1.

### Effects of Seasonality

**Figure 5:**
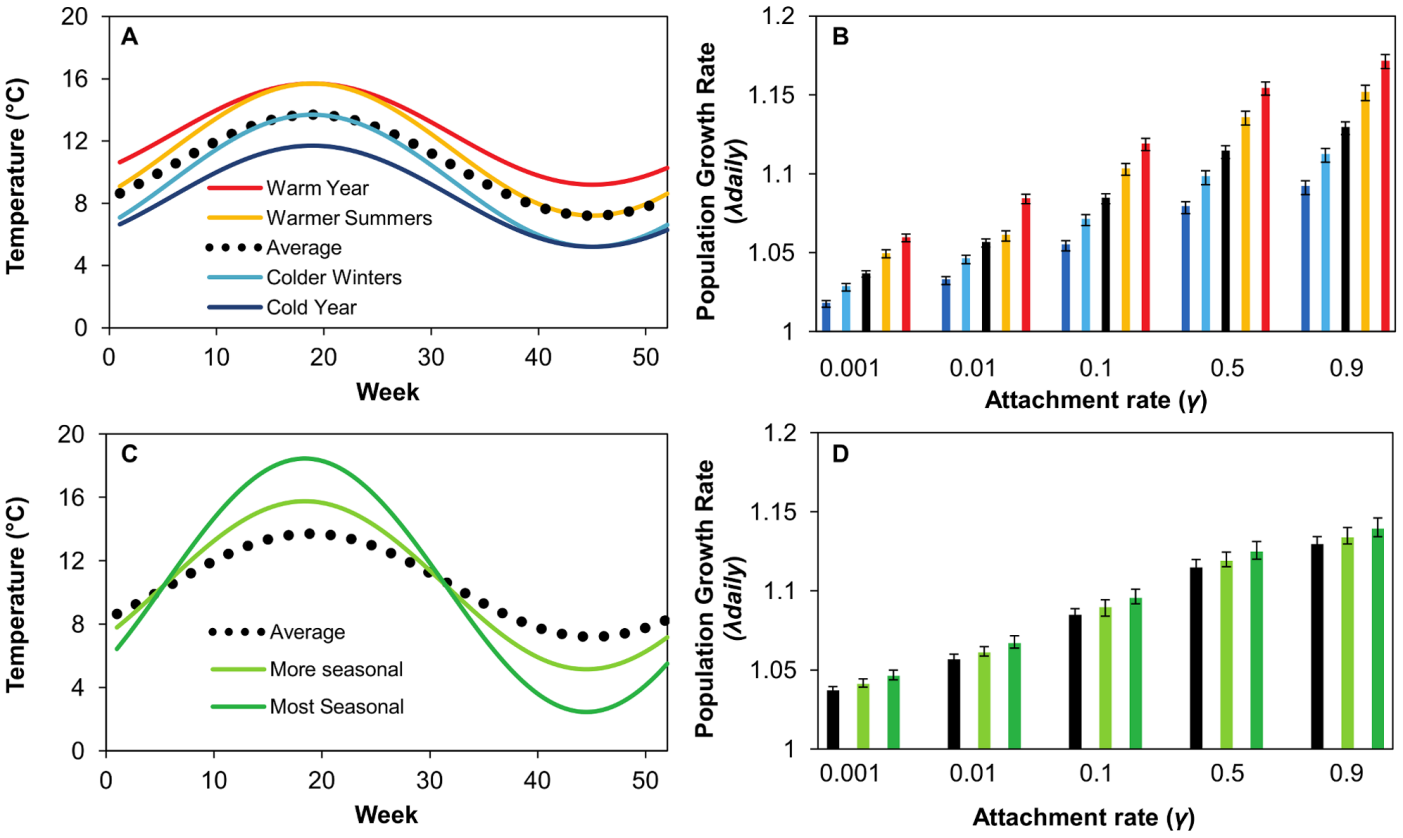
Temperature profiles used for population projection matrices their effect on λ_daily_. Minimum and maximum seasonal temperatures were altered relative to a baseline temperature (A) and temperature variance was increased relative to a baseline temperature (C). The daily population growth rate for each of these scenarios is shown (B and D). Baseline temperatures are averages of five years of temperature data from 33 farm sites in Scotland [53]. For all parameters means ± 95% confidence intervals are shown.

Both increases in yearly mean temperature and yearly temperature variation caused an increase in λ_daily_ (Figure 5A and 5B); however increases in yearly mean temperatures had a greater effect. For example, when *γ* = 0.1 per day, a 2°C increase in the yearly mean temperature caused estimates of λ_daily_ to increase from 1.085 to 1.119, while a 2°C decrease in the yearly mean temperature caused λ_daily_ to decrease to 1.055. Increasing the extreme values for one season caused a similar change. A 2°C decrease in the winter minimum caused λ_daily_ to decrease to 1.072, while a 2°C increase in the summer maximum temperature by 2°C caused λ_daily_ to change to 1.103 . Increasing the variation in temperature across the year also caused λ to increase (Figure 5C and 5D). When *γ* = 0.1, λ_daily_ increased to 1.089 in the more seasonal temperature profile and 1.096 in the most seasonal temperature profile.

For all temperature profiles, increasing the larval attachment rate caused λ_daily_ to increase. For example in the average seasonal temperature profile, λ_daily_ is 1.037, 1.057, 1.085, 1.114 and 1.130 for attachment rates of 0.001, 0.01, 0.1, 0.5 and 0.9 respectively.

### Effects of Treating Different Life Stages Across Seasons

**Figure 6:**
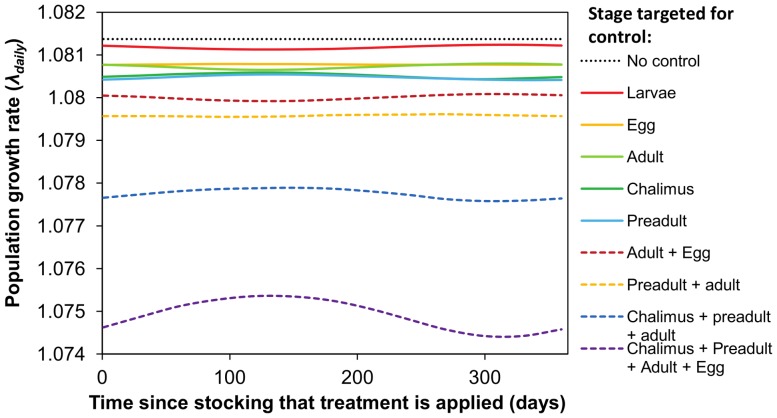
Effect of delivering a single control treatment at different times of the year on the population growth rate (λ_daily_) of sea lice. The y-axis indicates the time of year since stocking that the single control treatment was delivered. All simulations assume a spring stocking of salmon smolts, so day 1 is May 1. A treatment efficacy of 95%, a larval attachment rate of 0.1 and the most seasonal temperature scenario were used for all calculations. Results for treatments applied to different life stages are shown.

Analysis of the effect of single stage-targeted treatments across time show that both the life stage targeted and the time of treatment application can influence the effect of a treatment on λ (Figure 6). Treatments targeting eggs, chalimus, preadults and adults are similarly effective in the summer; however, in the winter treatments targeting chalimus and preadults are most effective. Treatment efficacy is far greater when several stages are targeted simultaneously.

## Discussion

Sea lice are a prominent marine ectoparasite that threaten the productivity of salmon farming and are associated with increased mortality in wild salmon [12], [13]. The matrix models presented here suggest that temperature can increase the rate at which infestations establish and develop on farmed salmonids as a result of increased reproductive success and development at high temperatures and because the dampening effect of mate limitation on population growth is more quickly overcome at higher temperatures. The life history of *L. salmonis* has been studied in much more detail than many other sea lice in the Caligidae family and construction of similar matrix models for other species of sea lice may not be possible due to the lack of data needed for parameterization [56]; however, many aspects of sea louse life history are similar across species including temperature-dependent development, high fecundity and the existence of a free-swimming larval stage that has an endogenous energy supply [39], [56]. Therefore it is likely that the temperature-dependent trends found in this study extend to other species in the Caligidae family.

Increased temperature causes more rapid development in sea lice for every life stage except copepodids, in which endogenous energy supplies provided to the egg and the probability of finding a host constrain survival and development [39]. One of the more dramatic results of increased development is the larger increase in net reproductive rate of sea lice. At high temperatures, they produce more surviving offspring in a shorter time than at low temperatures. Collectively these increases in development and fitness drive the rapid increase in population growth that occurs with increasing sea surface temperature.

The temperature ranges addressed in this study are within the ranges experienced on farms. Typical sea surface temperatures on salmon farms range from 1–14°C in Atlantic Canada, 6–18°C in Ireland and 1–20°C in some Norwegian fjords [49]; however, the majority of research on sea lice is focused on relatively moderate temperatures (e.g. between 6°C and 14°C). This study suggests that extreme temperatures are critical for determining the growth rate of a population. In addition, the wide confidence intervals for generation time and R_0_ at 4°C and in population growth at 20°C suggests that stochastic effects are more dominant at extreme temperatures and population trajectories at these temperatures may be more challenging to predict. The effect of extreme temperatures depends not only on their specific value, but on the entire profile of the seasonal variation. Increased overall variation does not have as large an effect on population growth as does an increase in mean temperature.

Despite the strong effect of temperature seen in this model and in laboratory studies [14], effects of temperature on sea louse infestations are not always detected in analyses of sea louse infestations in the field. Two studies conducted on field data of sea louse infestations on farms in Norway [17], [57] and one study conducted in British Columbia [49] found that temperature was positively correlated with sea louse abundance on farmed and wild sea trout. In contrast, other studies, conducted in British Columbia [58], Scotland [16] and Norway [15] found no detectable effect of temperature on sea louse abundances. One explanation for this apparent discrepancy may be that seasonal temperature decreases in the winter masked the effects of warmer summer temperatures. In addition, other factors, such as the attachment rate of copepodids may be influencing infestations more than temperature. This study suggests that, in order to detect a temperature effect on sea louse infestations, both the mean and the range of temperatures across a year must be considered.

Mate limitation is also influential in determining the rate at which a new population of sea lice increases. Mate limitation is especially pronounced at cold temperatures, during which more than a year may be required for new populations to reach abundances where mate limitation does not occur, if this threshold is reached at all. We made many assumptions about sea louse mating that require further research to quantify empirically. In particular, the assumptions of high promiscuity among males and females, equal sex ratios and co-aggregation of males and females all can dramatically influence mating success of parasites [45] and are not well quantified for sea lice [47]. In addition, host switching among preadult males is predicted to be density-dependent and may influence sex ratios and aggregation [59]. Skewed sex ratios have been found in some sea louse populations [60], but not others [47] and more work is needed to quantify this variation, identify underlying environmental and biological factors, and predict the consequences of this variation for population demographics.

While high louse abundances may cause negative density-dependent effects (i.e., over-compensation), we did not include these effects in our model. Over-compensation could occur if population growth is reduced when sea louse abundances on the host exceed thresholds above which immune responses in the host may limit attachment by the parasite (for example in *O. gorbuscha* [61]), or as a result of mortality of the infested host; however, there is limited evidence that Atlantic salmon exhibit effective immune responses against sea lice [37]. Moreover, it is unclear whether morbidity and mortality of salmon with high levels of infestations (e.g., mean annual levels greater than 20–30 sea lice per salmon) will feed back to influence the epidemiology of sea lice [59], [60].

The numerous methods used to control sea louse infestations on salmon farms must balance the negative effects of control strategies including costs [18], stress to salmon [23], environmental impacts of treatments [24], and evolution of drug resistance [20] against the positive result of sea louse control. The analyses performed in this study may inform specific recommendations for effective sea louse control. Elasticity analyses and analyses of single treatments in this study suggest that to be most effective, treatments for sea lice must target more than one stage. While all of the simulated control treatments reduced the population growth rate, none of them reduced lambda to levels lower than 1, at which point the population would start declining. This suggests that several treatments throughout the year is ideal for effective control in most situations. Treatments targeting chalimus and preadults are most effective at low temperatures and when the attachment rate of larvae is low. All treatments are less effective at controlling sea lice in the summer. Because we did not incorporate immigration of free-swimming copepodids into the model, and this can be an important source of sea lice in some locations [42], it is also possible that we are underestimating the role that this stage may play in areas with high connectivity between farms.

Matrix elements with the highest sensitivities are most likely to influence fitness (λ) and evolutionary responses (i.e. drug resistance) may emerge faster when these stages are targeted for control. If heritable variation for resistance exists in selected sea louse populations, resistance to treatments may evolve more quickly when treatments are targeting the preadults. This is particularly relevant as there is interest in developing immunostimulants that may increase the ability of the host to reject attaching copepodids [62]. Such a control strategy has the potential to be successful because sea lice may be less likely to evolve resistance.

In addition to specific suggestions for more strategic use of treatments in response to sea louse monitoring, the temperature-dependent PPMs in this study give some insight into the potential for climate change to exacerbate sea louse infestations. While the level of uncertainty in projected oceanic conditions is high, many climate change models predict increases in temperate sea surface temperatures as a result of increased stratification, decreased upwelling and altered circulation [63]. However, increased temperature is only one component of projected changes to marine environments; oceans are predicted to experience sea-level rise, altered circulation, decreased salinity and decreased pH [63]. These other factors have the potential to influence sea louse epidemiology; increased circulation may decrease the attachment rate of infectious copepodids by transporting them away from susceptible hosts [64] and decreased salinity decreases attachment of copepodids [50], [65]. Further studies are needed to understand the net effects of the projected oceanic conditions on geographical ranges and potential of sea louse epizootics on both farmed and wild salmon. In addition, more work is needed to parameterize sea louse life history at high and low temperature extremes. For example, a maximum temperature threshold for *L. salmonis* has not been determined and it is possible that physiological costs of thermotolerance may decrease survival or fitness of sea lice at high temperatures [56].

More work is necessary to understand the effects of temperature variation on wild salmon. Increased temperature means and variation are predicted to increase sea louse infestations on farmed salmon, suggesting that careful consideration of temperature must be taken into account when sites for salmon farming are chosen, especially in areas that may be predisposed to high levels of exposure to sea lice due to hydrodynamic conditions and proximity to existing salmon farms or wild salmon migration routes. In contrast to salmon farms, the densities and locations of wild salmon fluctuate temporally and the costs of infestations may be mediated by other stressors that are less likely to occur on salmon farms (for example, food limitation and predation) [66]. Quantifying spatial movement and physiological states of wild salmon in relation to their sea lice exposure is an enormous challenge, but may be a major benefit to understanding and reducing conflicts between wild salmon conservation and salmon farming.

## Conclusions

The model built in this study differs from many previously constructed agent-based and system-dynamic models of sea louse population dynamics; its’ greatest utility is that it can be used to highlight specific characteristics of the life history of this ectoparasite that greatly influence its population growth. As such the results of this study may be informative for designing management programs to reduce the potential for sea louse epidemics especially when taking seasonal and inter-annual temperature variation into account. The broad recommendations that are generated by this study support the continued use of matrix population models for understanding the epidemiology of ectoparasites from a life history perspective.

## Supporting Information

Appendix S1 **Population projection matrices (means and standard deviations) of sea lice at 4°C, 8°C, 12°C, 16°C and 20°C.**
(DOC)

Appendix S2 **Matrices of sea lice sensitivities (means and standard deviations) at 4°C, 8°C, 12°C, 16°C and 20°C and for attachment rates of 0.001, 0.01, 0.1, 0.5 and 0.9.**
(XLSX)

Appendix S3 **Matrices of sea lice elasticities (means and standard deviations) at 4°C, 8°C, 12°C, 16°C and 20°C and for attachment rates of 0.001, 0.01, 0.1, 0.5 and 0.9.**
(XLSX)

Appendix S4 **Stable stage equilibrium and time to equilibrium.**
(DOC)

Code S1 **Instructions on the use of the annotated R-code.**
(R) Click here for additional data file.

Code S2 **R-code for matrix model with fixed temperatures.**
(R) Click here for additional data file.

Code S3 **R-code for matrix population models with seasonal variation.**
(R) Click here for additional data file.

Code S4 **R-code for matrix population models with seasonal variation and treatments.**
(R) Click here for additional data file.

Code S5 **R-code for matrix population project with density-dependent mating.**
(R) Click here for additional data file.

Code S6 **R-code for parameters in the matrix model.**
(R) Click here for additional data file.

Code S7 **R-code for parameters in the matrix model when there is density-dependent mating.**
(R) Click here for additional data file.

Code S8 **R-code for the matrix A.**
(R) Click here for additional data file.
