# Supplementary material for: Modelling the Impact of Temperature-Induced Life History Plasticity and Mate Limitation on the Epidemic Potential of a Marine Ectoparasite
Source: PLoS One. 2014 Feb 5;9(2):e88465. doi: 10.1371/journal.pone.0088465 (PMC3914972; doi:10.1371/journal.pone.0088465)
Supplement: Appendix S1 — Population projection matrices (means and standard deviations) of sea lice at 4°C, 8°C, 12°C, 16°C and 20°C. (DOC) [file pone.0088465.s001.doc]

**Supporting Information 1**

Population projection matrices of sea lice (*Lepeophtheirus salmonis*) determined for fixed temperatures are shown below. For each entry, means are shown above and standard deviations are in parentheses below. Matrices Terms *P_2_* and *G_2_* are dependent upon the attachment rate of copepodids to the host (*γ*). Values for these terms are shown below the matrix. The parameter
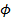
 varies between 0 and 1 as a function of the abundance of adult female lice per host (equations 9 and 10) and is used in calculations where density dependent mating is considered. Values are based on 1000 calculations of the population projection matrix.

where *P_2_* = 0.183 ± 0.002, 0.300 ± 0.0006, 0.488 ± 0.002, 0.684 ± 0.003 , 0.772 ± 0.004 and *G_2_* = 0.0107 ± 0.001, 0.0177 ± 0.002, 0.0293 ± 0.003, 0.041 ± 0.005, 0.047 ± 0.005 for *γ* = 0.001, 0.01, 0.1, 0.5 and 0.9 respectively.

where *P_2_* = 0.181 ± 0.0002, 0.295 ± 0.0004, 0.477 ± 0.001, 0.661 ± 0.002 0.743 ± 0.003 and *G_2_* = 0.0191 ± 0.0008, 0.0315 ± 0.001, 0.0520 ± 0.002, 0.0738 ± 0.003, 0.0840 ± 0.004 for *γ* = 0.001, 0.01, 0.1, 0.5 and 0.9 respectively.

where *P_2_* = 0.180 ± 0.00009, 0.292 ± 0.0002, 0.469 ± 0.0007, 0.644 ± 0.001, 0.721 ± 0.002 and *G_2_* = 0.0252 ± 0.0005, 0.0416 ± 0.0009, 0.0687 ± 0.001, 0.0974 ± 0.002, 0.111 ± 0.002 for *γ* = 0.001, 0.01, 0.1, 0.5 and 0.9 respectively.

where *P_2_* = 0.180 ± 0.00008, 0.290 ± 0.0002, 0.463 ± 0.0006, 0.633 ± 0.001, 0.697 ± 0.002 and *G_2_* = 0.0293 ± 0.0004, 0.0484 ± 0.0008, 0.0799 ± 001, 0.113 ± 0.002, 0.128 ± 0.002, for *γ* = 0.001, 0.01, 0.1, 0.5 and 0.9 respectively.

where *P_2_* = 0.179 ± 0.00007, 0.29 ± 0.0002, 0.459 ± 0.0005, 0.625 ± 0.001, 0.697 ± 0.001 and *G_2_* = 0.0321 ± 0.0004, 0.0529 ± 0.0007, 0.0873 ± 0.001, 0.124 ± 0.002, 0.141 ± 0.002, for *γ* = 0.001, 0.01, 0.1, 0.5 and 0.9 respectively.
