# Supplementary material for: Modelling the Impact of Temperature-Induced Life History Plasticity and Mate Limitation on the Epidemic Potential of a Marine Ectoparasite
Source: PLoS One. 2014 Feb 5;9(2):e88465. doi: 10.1371/journal.pone.0088465 (PMC3914972; doi:10.1371/journal.pone.0088465)
Supplement: Appendix S4 — Stable stage equilibrium and time to equilibrium. (DOC) [file pone.0088465.s004.doc]

**Supporting Information 4**

Analyses of vital rates based on population projection matrices assume that a population is in stable stage equilibrium. When colonizing a new population of hosts, sea lice are not in equilibrium. In order to determine how long it takes to reach equilibrium states, we simulated each of the scenarios described above for 100 days in order to observe the time until the proportion of individuals in each stage was stable. For these simulations the initial population was seeded with 10 larvae. We also calculated the proportion of individuals in each lifestage at equilibrium by calculating the left eigenvector of the matrix.

The time for populations to reach stable stage equilibriums decreased with increases in temperature and copepodid attachment rate (Fig. S1). Populations reached equilibrium values within 20 days at 20⁰ C regardless of the copepodid attachment rate, but took as long as 60 (when *γ* = 0.5) or 80 days (when *γ* = 0.001) at 4⁰ C. Temperature and the copepodid attachment rate also affected the proportions of individuals in each life stage when the population reached equilibrium. Both temperature and the copepodid attachment rate caused the proportion of chalimus to increase and the proportion of preadults to decrease (Fig. S2). Increases in temperature caused the proportion of adult males and females in the population to decrease.


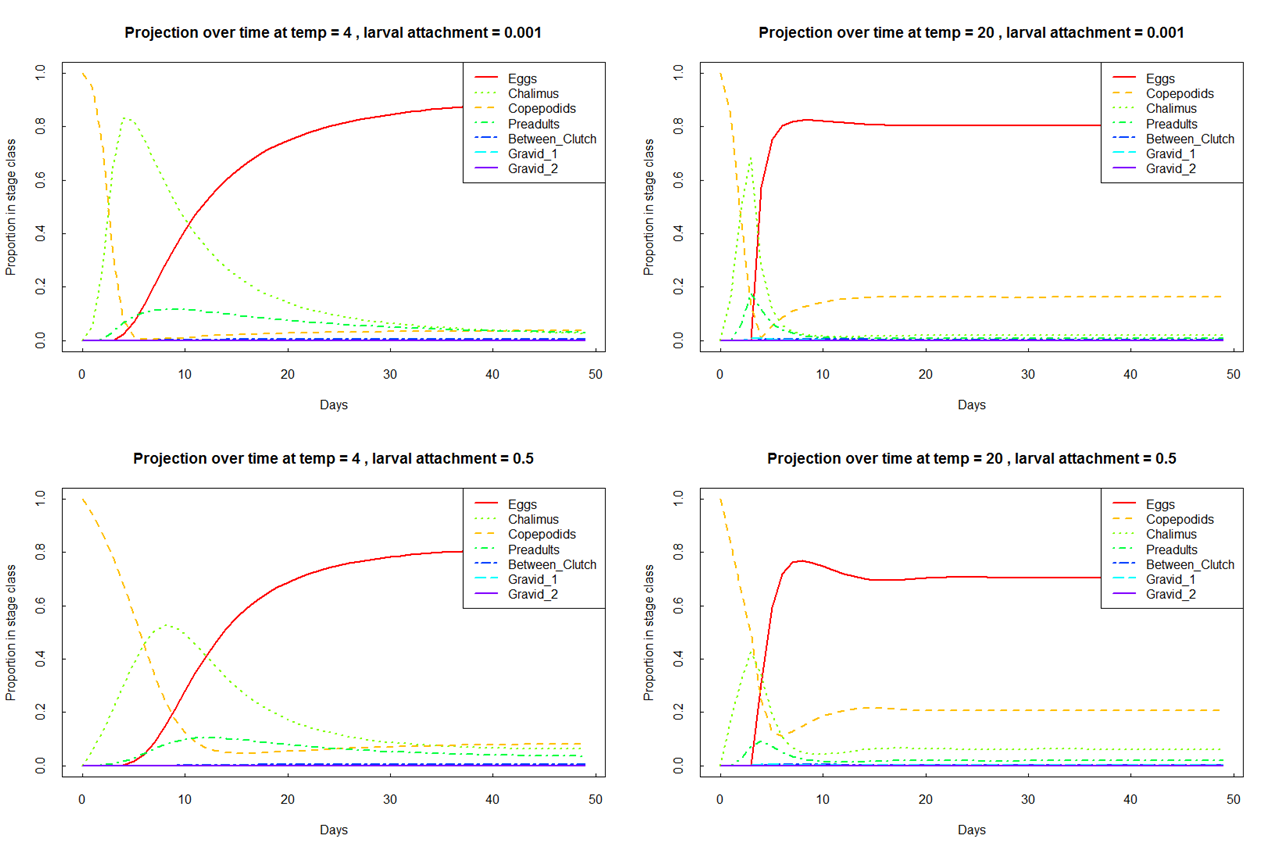


**Figure S1. Proportion of individuals in stage classes over time.** Data are for 4⁰ C and 20⁰ C and for copepodid attachment rates of 0.001 and 0.5.


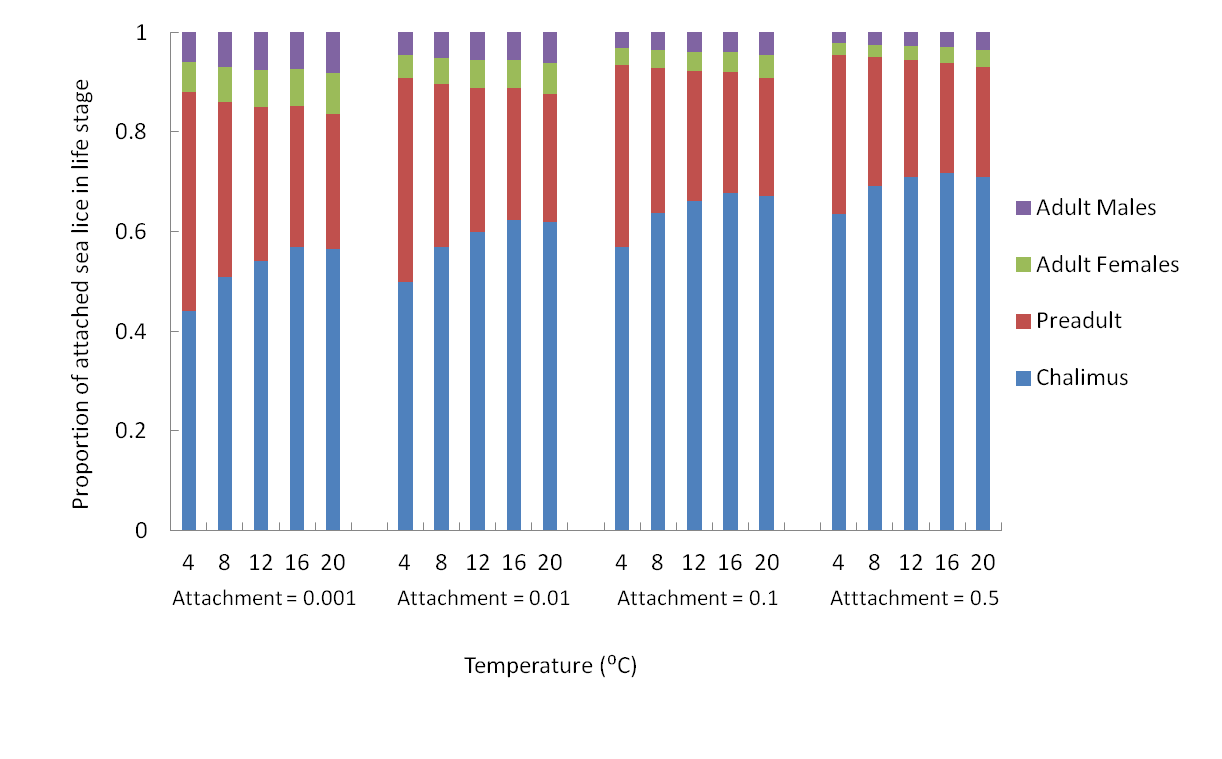


**Figure S2. Proportion of individuals in each attached life history stage that is attached to the host.** Data shown for various temperatures and copepodid attachment rates.
